# SARS-CoV-2 Spike Protein Destabilizes Microvascular Homeostasis

**DOI:** 10.1128/Spectrum.00735-21

**Published:** 2021-12-22

**Authors:** Soumya Panigrahi, Tamal Goswami, Brian Ferrari, Christopher J. Antonelli, Douglas A. Bazdar, Hannah Gilmore, Michael L. Freeman, Michael M. Lederman, Scott F. Sieg

**Affiliations:** a Division of Infectious Diseases and HIV Medicine, Case Western Reserve University School of Medicine, Cleveland, Ohio, USA; b Department of Chemistry, Raiganj University, Raiganj, West-Bengal, India; c Department of Pathology, Case Western Reserve University School of Medicine, Cleveland, Ohio, USA; Kumamoto University

**Keywords:** COVID-19, spike protein, endothelial cells

## Abstract

SARS-CoV-2 infection can cause compromised respiratory function and thrombotic events. SARS-CoV-2 binds to and mediates downregulation of angiotensin converting enzyme 2 (ACE2) on cells that it infects. Theoretically, diminished enzymatic activity of ACE2 may result in increased concentrations of pro-inflammatory molecules, angiotensin II, and Bradykinin, contributing to SARS-CoV-2 pathology. Using immunofluorescence microscopy of lung tissues from uninfected, and SARS-CoV-2 infected individuals, we find evidence that ACE2 is highly expressed in human pulmonary alveolar epithelial cells and significantly reduced along the alveolar lining of SARS-CoV-2 infected lungs. *Ex vivo* analyses of primary human cells, indicated that ACE2 is readily detected in pulmonary alveolar epithelial and aortic endothelial cells. Exposure of these cells to spike protein of SARS-CoV-2 was sufficient to reduce ACE2 expression. Moreover, exposure of endothelial cells to spike protein-induced dysfunction, caspase activation, and apoptosis. Exposure of endothelial cells to bradykinin caused calcium signaling and endothelial dysfunction (increased expression of von Willibrand Factor and decreased expression of Krüppel-like Factor 2) but did not adversely affect viability in primary human aortic endothelial cells. Computer-assisted analyses of molecules with potential to bind bradykinin receptor B2 (BKRB2), suggested a potential role for aspirin as a BK antagonist. When tested in our *in vitro* model, we found evidence that aspirin can blunt cell signaling and endothelial dysfunction caused by bradykinin in these cells. Interference with interactions of spike protein or bradykinin with endothelial cells may serve as an important strategy to stabilize microvascular homeostasis in COVID-19 disease.

**IMPORTANCE** SARS-CoV-2 causes complex effects on microvascular homeostasis that potentially contribute to organ dysfunction and coagulopathies. SARS-CoV-2 binds to, and causes downregulation of angiotensin converting enzyme 2 (ACE2) on cells that it infects. It is thought that reduced ACE2 enzymatic activity can contribute to inflammation and pathology in the lung. Our studies add to this understanding by providing evidence that spike protein alone can mediate adverse effects on vascular cells. Understanding these mechanisms of pathogenesis may provide rationale for interventions that could limit microvascular events associated with SARS-CoV-2 infection.

## INTRODUCTION

The corona virus disease 2019 (COVID-19) pandemic has severely affected millions of people across the globe. Although many individuals infected with severe acute respiratory syndrome coronavirus-2 (SARS-CoV-2) develop a mild or moderate infection, 15–20% of patients experience severe pneumonia that may progress to respiratory distress and eventually multi-organ failure. SARS-CoV-2 uses the transmembrane glycoprotein angiotensin-converting enzyme II (ACE2) for entry into host cells (1, 2). The transmembrane glycoprotein ACE2 is present on vascular and cardiac endothelial cells, ciliated columnar epithelial cells of the upper respiratory tract, sparsely on Type-1, and markedly on the Type-2 alveolar epithelial cells (3, 4). The cellular transmembrane serine protease 2 (TMPRSS2) is also needed to prime the SARS-CoV-2 spike protein of the virus before its cellular entry (5). Virus entry may also depend on activity of the endosomal/lysosomal cysteine proteases (cathepsins B or L) although their activity is likely dispensable (6).

In addition to permitting viral entry, the interaction of ACE2 with SARS-CoV2 spike protein reduces ACE2 cell surface expression and impairs function. ACE2 is a carboxypeptidase that cleaves angiotensin-1 (AT-1) and angiotensin-2 (AT-2), and physiologically functions as a homeostatic regulator of blood pressure (7). ACE2 is also one of the inactivating proteases responsible for neutralizing about 80–90% of the circulating bradykinin (BK) in the pulmonary vascular bed (8). Excess BK potentially facilitates capillary dilation, leaking of protein rich exudates, and organ failure (9). In inflammatory settings, BK engages its dedicated receptors, disrupting the capillary endothelial barrier function and facilitating the entry of immune cells into pulmonary interstitial tissue space (10, 11). In addition, SARS-CoV-2 increase the activity of renin angiotensin system (RAS), elevates AT-2 levels, and decrease ACE2 mRNA expression in postmortem samples (12, 13). Thus, a reduction in ACE2 potentially dysregulate both AT-2 and BK homeostasis contributing to pathological changes in the respiratory tract (14). Despite an appreciation for the potential implications of spike/ACE2 interactions in SARS-CoV-2 pathogenesis, detailed understanding about the impact of SARS-CoV-2 engagement to the host at the cellular and molecular level is far from complete.

In COVID-19, viral RNA has been localized to cells of the conducting airways and alveoli by *in situ* hybridization in postmortem samples (15). Here, we examined ACE2 and BK receptor B2 (BKRB2) expression *in vivo* to appreciate the distribution of these receptors in the respiratory tract and to assess ACE2 expression in the lining of the alveoli, comparing postmortem lung tissues from COVID-19 patients to those of uninfected controls. Using *in vitro* approaches, we confirmed the expression of ACE2 and BKRB2 in primary human cell types and we interrogated the consequences of spike protein and BK interactions with both primary alveolar epithelial and endothelial cells. Our observations suggest that SARS-CoV-2 spike protein diminishes ACE2 expression in these cell types. In addition, we observed that exposure to SARS-CoV-2 spike protein down modulate Krüppel-like Factor 2 (KLF2) expression, induces caspase activation, and apoptosis in human aortic endothelial cells. Moreover, exposure of endothelial cells to BK also promotes endothelial dysfunction and this effect can be suppressed by aspirin or other potential BKRB2 antagonists. Thus, we propose that the diminished ACE2 level is caused in part by direct interactions with SARS-CoV-2 spike protein but also by cytopathic effects of the virus on ACE2 expressing Type-2 alveolar epithelial cells, and vascular endothelial cells. Such effects may be associated with local release of pro-inflammatory cytokines, detailed in (16). These observations provide clues for deciphering mechanisms which SARS-CoV-2 utilizes to initiate development of acute respiratory distress syndrome (ARDS), leading to poorly manageable respiratory failure. Our observations offer rationale for recommending explorative targeted BKRB2 blocking strategies at different stages of COVID-19 pathogenesis (17, 18).

## RESULTS

### Diminished ACE2 expression in alveolar lining of COVID-19 patients.

**FIG 1:**
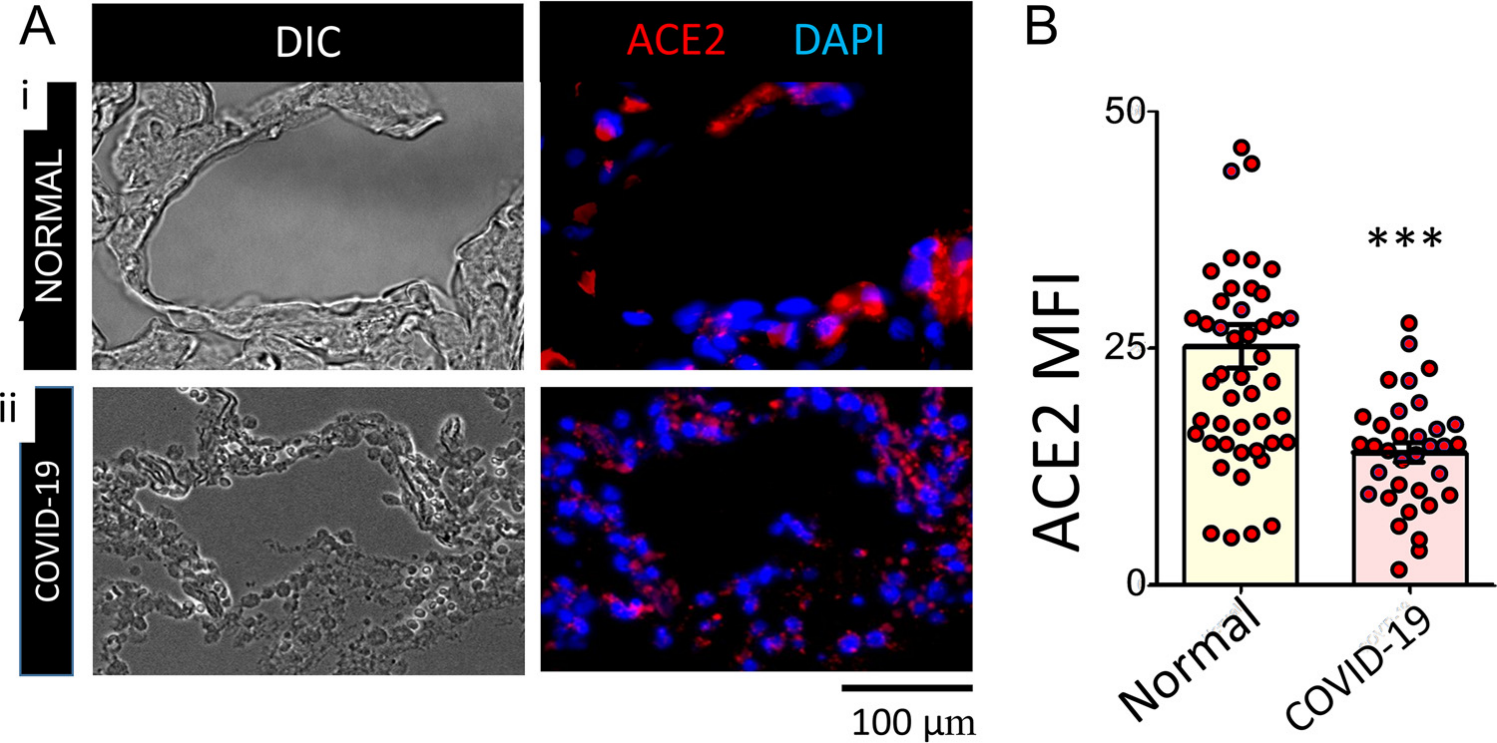
Surface expression of ACE2 in normal and COVID-19 lung alveoli. (A): Representative DIC (BW), ACE2/DAPI (red/blue) epi-fluorescence microscopy images and mean fluorescence intensity (MFI) of ACE2 from indicated numbers of healthy (A-i), and SARS-CoV-2 (A-ii) lung FFPE sections shown in the upper and lower rows respectively. (B): Quantified MFI data of ACE2 levels in normal and COVID-19 lung alveoli (*n* = 10 + 4; ***, *p* < 0.001).

We studied autopsy lung tissues from de-identified uninfected donors and SARS-CoV-2 infected individuals (Table S1 in the supplemental material). Data presented in Fig. 1A shows ACE2 expression in representative immunofluorescence images of normal and COVID-19 lung tissues. The relative ACE2 expression measured by mean fluorescence intensity (MFI) values in normal and SARS-CoV-2 infected tissue, indicated a significant reduction of ACE2 along the alveolar lining in lungs of COVID-19 patients compared to findings among controls (Fig. 1B).

Recent transcriptional studies point identify type II pneumocytes as the primary cell population expressing ACE2 in the lower respiratory tract (19). To better understand the cell types expressing ACE2 in alveoli, we examined ACE2 expression in Type-2 alveolar epithelial cells in normal lung tissue by immunostaining ACE2 and surfactant protein C (spC). As anticipated, representative composite images indicate that the spC-expressing cells in the alveolar wall also express ACE2; nonetheless, there are also cells not expressing spC in the alveolar/peri-alveolar regions with strong expression of surface ACE2 (Fig. S1A in the supplemental material). We further determined the specificity of ACE2 expression in cultured, primary alveolar epithelial cells, which also co-express spC (Fig. S1B-i). In Fig. S1B-ii representative high-resolution fluorescence microscopy images from isotype control IgG stained primary alveolar epithelial cells were shown indicating the high stringency of these immunofluorescence staining.

We also tested the ACE2 expression on human primary aortic endothelial cells in our system. Briefly, endothelial cells were grown on glass slides and immunofluorescence staining was done on nonpermeabilized cells targeting surface ACE2. Representative image data in Fig. S1C-i, shows surface expression of ACE2, on endothelial cells. Respective images immunostained with isotype control antibodies in identical conditions from the same set of experiments are shown in Fig. S1C -ii.

We hypothesize that increased BK activity due to reduced ACE2 expression could play a role in COVID-19 pathogenesis. Therefore, we assessed ACE2 and BKRB2 expression throughout the respiratory airway epithelium using normal lung autopsy samples from SARS-CoV-2-uninfected subjects. The diagram in Fig. S2A in the supplemental material provides a schematic representation of the respiratory tract and theoretical distribution of ACE2 and BKRB2 in the alveolar space. Figure S2B shows the distribution of alveolar epithelial cells surface expressions of ACE2 and BKRB2. Moreover, in Fig. S2B (i-iv) composite immunofluorescence images are shown for larynx (*n* = 8), trachea (*n* = 6), bronchus (*n* = 4), and alveoli (*n* = 10) from normal lung (S2B i-iv). Overall, we found evidence that ACE2 and BKRB2 are expressed on alveolar epithelial cells and vascular endothelial cells generally appears at close proximity in the respiratory tract (Fig. S2B).

### SARS-CoV-2 spike protein modulates surface expression of ACE2 on endothelial cells, alveolar epithelial cells and induces endothelial cell dysfunction.

**FIG 2:**
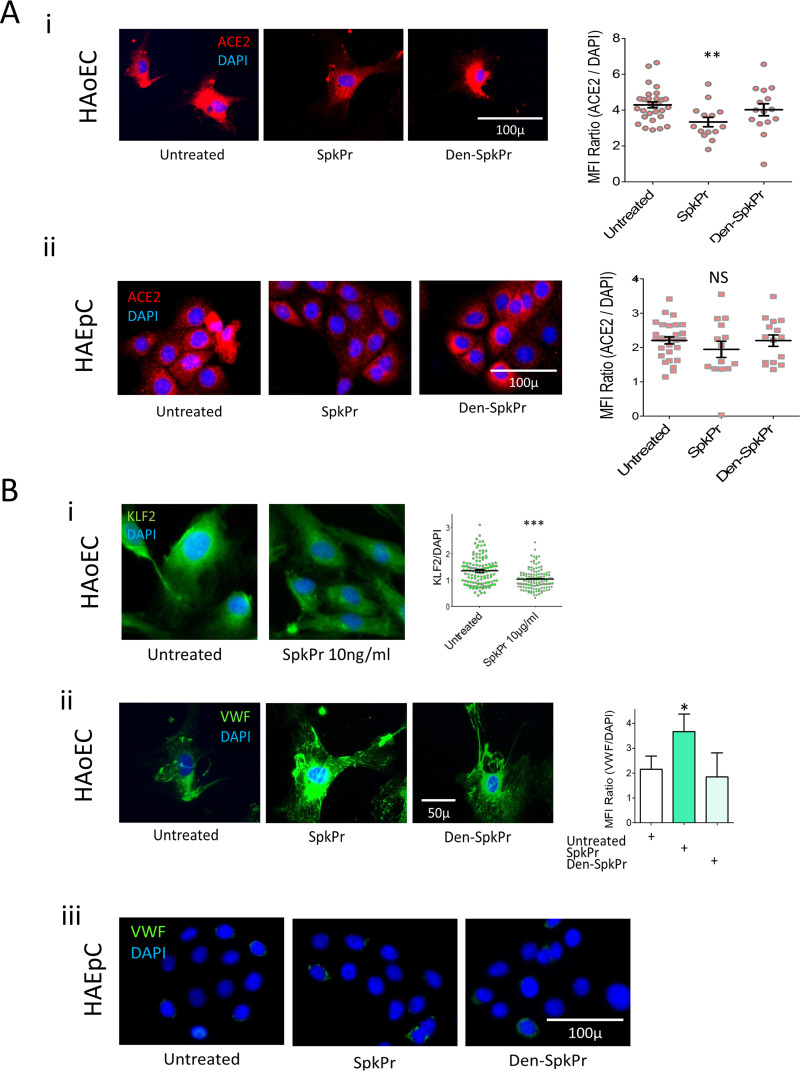
Effects of SARS-CoV-2 spike protein (SpkPr) on primary human aortic endothelial cells (HAoEC) and alveolar epithelial cells (HAEpC). (A-i, ii) Representative images and relative MFI/DAPI data of surface ACE2 on HAoEC and HAEpC, after 24h exposure to SpkPr (10 ng/ml) and respective controls. (B-i) Krüppel-like Factor 2 (KLF2) levels presented as normalized values of KLF2/DAPI fluorescence following 24h exposure of SpkPr (10 ng/ml) and B-ii surface vWF levels following 24h exposure of SpkPr (10 ng/ml) and respective controls—untreated or Den-SpkPr. (B-iii) Control experiment showing nominal surface vWF expression following SpkPr treatment. (*n* = 300+ cells; NS, not significant; *, *P* < 0.05; **, *P* < 0.005; ***, *P* < 0.001).

Previous studies suggest that ACE2 is shed from the surface of pulmonary epithelial and endothelial cells after spike protein interactions (reviewed in Ref. 14). We asked if ACE2 expression is reduced in a similar manner from the surface of nonpermeabilized primary human aortic endothelial cells (HAoEC) and primary human alveolar epithelial cells (HAEpC) *ex-vivo.* We also examined indices of dysfunctional changes in the endothelial cells *in vitro*. Primary HAoEC (Fig. 2A-i) and HAEpC (Fig. 2A-ii) cells were incubated with recombinant spike protein for 24 h, and surface expression of ACE2 (Fig. 2A-ii), intracellular expression of KLF2 (Fig. 2B-i) and surface expression of von Willebrand factor (vWF) HAoEC (Fig. 2B-ii), were analyzed using epi-fluorescence microscopy. Overnight exposure of these cells to spike protein (SpkPr; 10 ng/ml) significantly reduced surface ACE2 on HAoEC surface (Fig. 2A-i); we also observed a similar trend of surface ACE2 reduction pattern in HAEpC receiving identical treatment (Fig. 2A-ii). Interestingly, no such reduction of ACE2 expressions were observed when parallel control experiments were done under identical conditions in using heat denatured spike protein (Den-SpkPr). When 1% formaldehyde or sodium-azide treated dead endothelial cells were exposed to spike protein in identical control experiments, the surface expression of ACE2 did not change, suggesting that interference of ACE2 antibody staining by Spike occupancy of ACE2 receptor is not a likely explanation for reduced ACE2 surface expression detected in these experiments (not shown).

Interestingly, incubation of HAoECs with spike protein caused reduced expression of transcription factor KLF2, and increased expression of the pro-coagulant, vWF, consistent with induction of endothelial dysfunction (Fig. 2B-i, ii). These effects were not observed when cells were incubated with denatured spike protein. In Fig. 2B-iii a set of control fluorescence image data are presented where HAEpC were immunostained for vWF after SpkPr or Den-SpkPr treatment indicating nominal fluorescence for vWF.

### SARS-CoV-2 spike protein induces caspase activation and apoptosis in primary human aortic endothelial cells.

**FIG 3:**
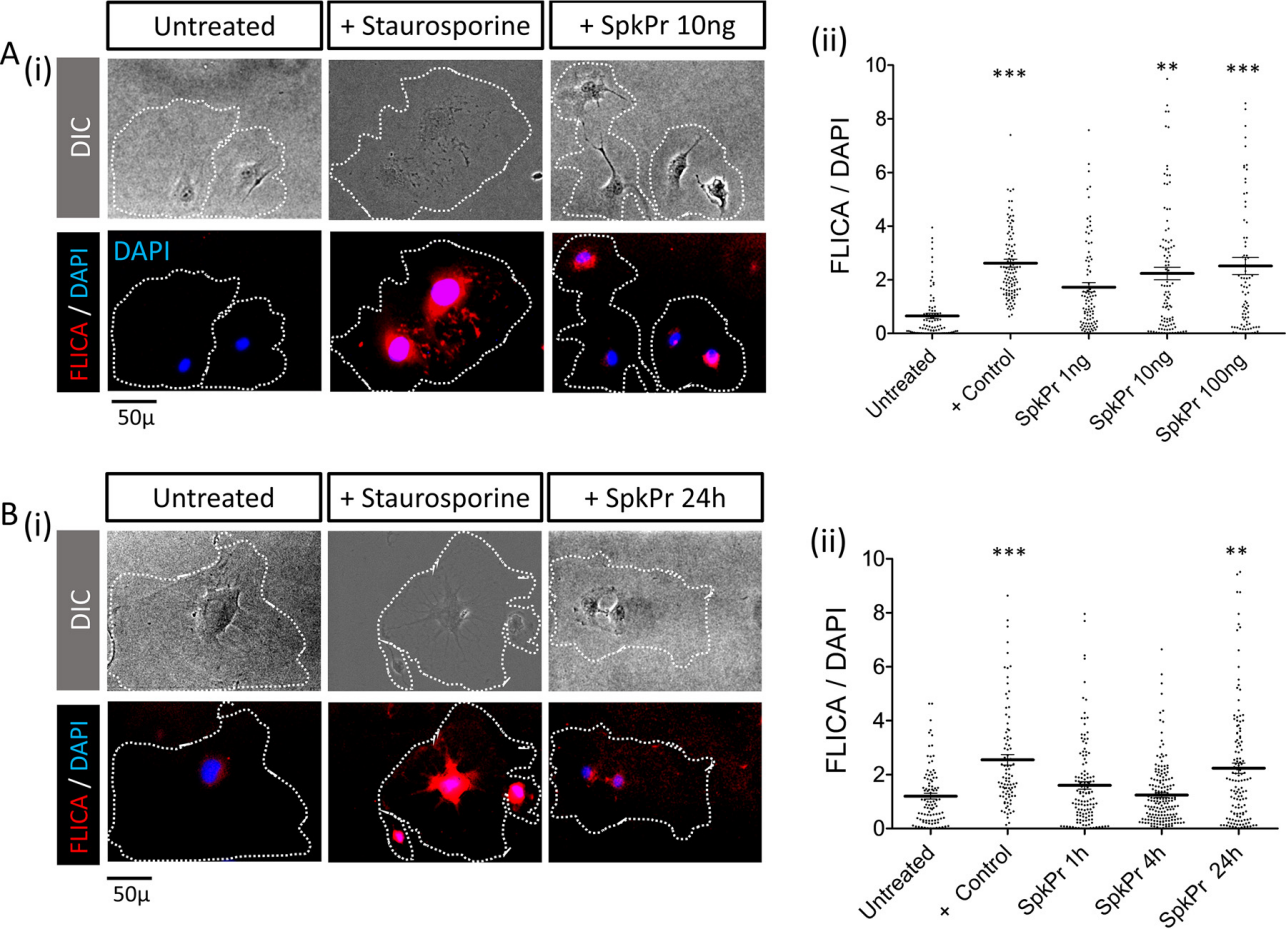
SARS-CoV-2 spike protein (SpkPr) induced pan-caspase activation in endothelial cells. (A-i) Representative SR-FLICA (red), DIC, and DAPI (blue) composite images of individual untreated, staurosporin (1 μM positive control), and SpkPr (1, 10, and 100 ng/ml −72h) treated endothelial cells, and (A-ii) respective normalized mean fluorescence intensity values. (B-i) Representative SR-FLICA (red), DIC, and DAPI (blue) composite images of individual untreated, staurosporin (1 μM −1 h positive control), and SpkPr 10 ng/ml (1, 4, and 24h) treated endothelial cells, and (B-ii) respective normalized mean fluorescence intensity values. (*n* > 100 cells, **, *P* < 0.005; ***, P < 0.001).

**FIG 4:**
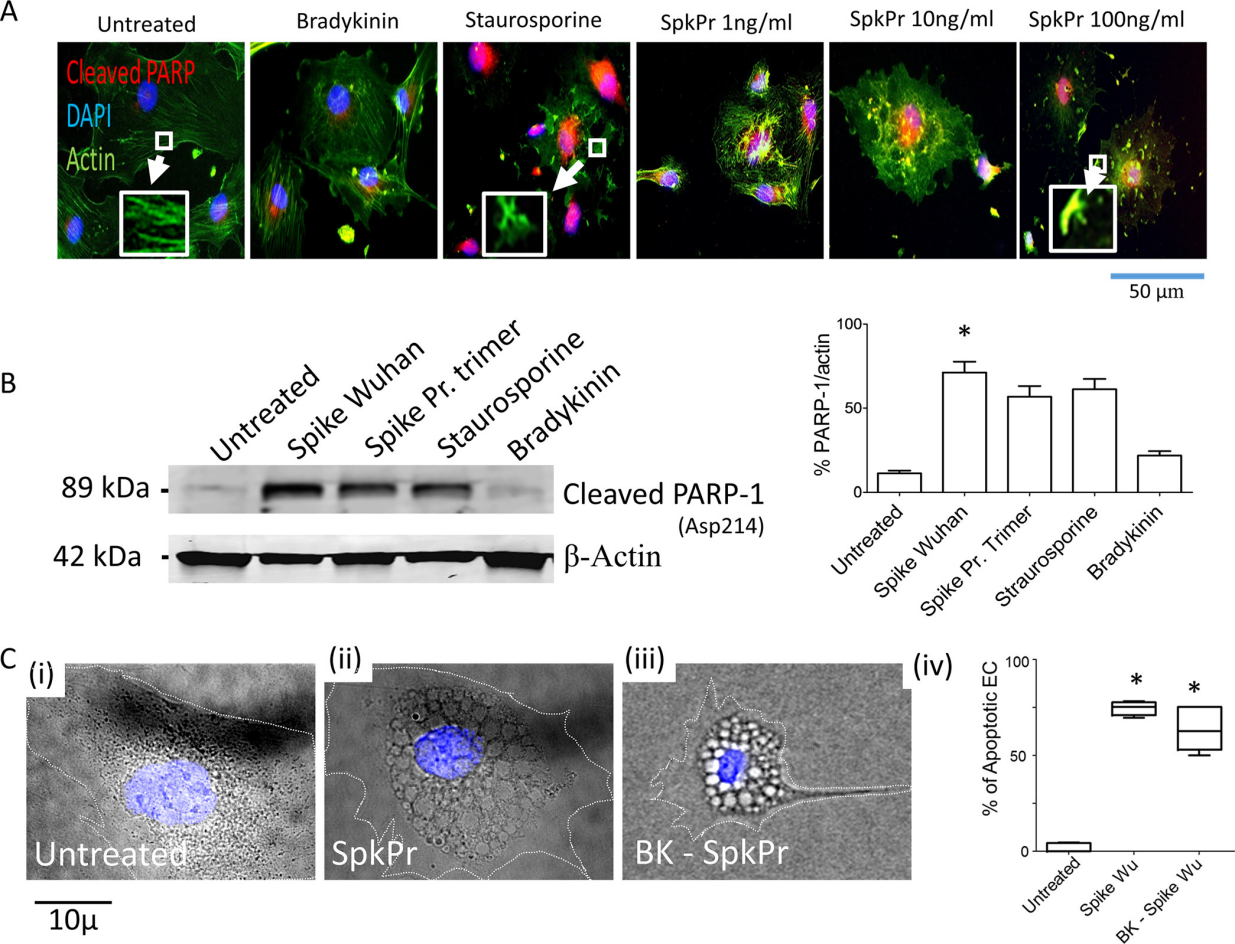
SARS-CoV-2 spike protein (SpkPr) and PARP-1 cleavage in endothelial cells. (A) Representative SR-FLICA (red), actin, and DAPI (blue) composite images of individual untreated, staurosporin (1 μM positive control), and SpkPr (1, 10, and 100 ng/ml) treated endothelial cells for 72 h (Inset: actin disassembly). (B) Representative Western blot data showing cleaved PARP-1(asp214) after 72 h of exposure of recombinant spike protein-Wuhan, & recombinant spike Pr. Active trimer (100 ng/ml, *n* = 3). (C-i–iv), Representative DIC and DAPI (blue) composite images of individual EC following indicated treatments. Percentage of morphologically apoptotic ECs after indicated treatments. (*n* > 100 cells, NS, not significant; *, *P* < 0.05).

To elucidate the intracellular signaling events in primary human aortic endothelial cells following ACE2 binding of spike protein, we measured poly-caspase activation using fluorochrome conjugated SR-FLICA reagent as per manufacturer’s instructions (https://immunochemistry.com/product/sr-flica-poly-caspase-assay-kit/) (20, 21). Endothelial cells (HAoECs) were exposed to SARS-CoV-2 spike protein for 24 h at concentrations of 0, 1, 10, 100 ng/ml (Fig. 3A-i–ii), and for the time dependence studies - endothelial cells were incubated to a fixed concentration of spike protein at 10 ng/ml for 1, 4, and 24 (Fig. 3B-i–ii). Some cells were exposed to staurosporine (1 μM) for 1 h as a positive control. We found evidence of concentration and time-dependent pan-caspase activation in HAoECs incubated with SARS-CoV-2 spike protein. To compare the effects of monomeric native spike protein versus proline stabilized trimeric protein, in subsequent studies, we incubated endothelial cells with Wuhan variant (Creative Biomart), and active trimeric spike protein (R&D Technologies) (100 ng/ml), in addition to untreated, BK (10 μM), and staurosporine (1 μM) treated control groups for 72 h. Compared to cells incubated in medium alone, there were enhanced levels of cleaved PARP-1 (Asp214) in the nuclei, disruption of actin filaments as indicated, by our immunofluorescence (Fig. 4A), and immunoblot data (Fig. 4B). We also observed significantly increased frequencies of apoptotic endothelial cells with characteristic morphological changes (cytoplasmic vacuoles, nuclear fragmentation, and detachment) (Fig. 4C-i–iv), in cells incubated with spike protein. Thus, extended exposure to SARS-CoV-2 spike protein induces apoptosis in primary human aortic endothelial cells.

### BK induced calcium signaling, and endothelial cell dysfunction can be decreased by aspirin *in vitro*.

We hypothesize that local effects of excess BK contribute to the SARS-CoV-2 induced rapid onset ARDS by acting on the peri-alveolar endothelium, which facilitate capillary dilation and fluid leak into the alveolar interstitial space (9, 22). Therefore, to assess the impact of vaso-active BK on the endothelium, we investigated the BK induced intracellular calcium redistribution pattern in the HAoECs. Initially we looked at the binding kinetics and affinity of BK, to the ligand-binding domain of BKRB2 that is robustly expressed on the surface of HAoECs (Fig. S3A). BKBR2, but not ACE2, was expressed on MRC-5 fibroblasts (Fig. S3B). As a control for the negative MRC-5 ACE2 staining, in Fig. S3C, we show representative images of intestinal epithelial CACO-2 cells that were stained in parallel for ACE-2 expression and indicate robust presence of ACE2.

**FIG 5:**
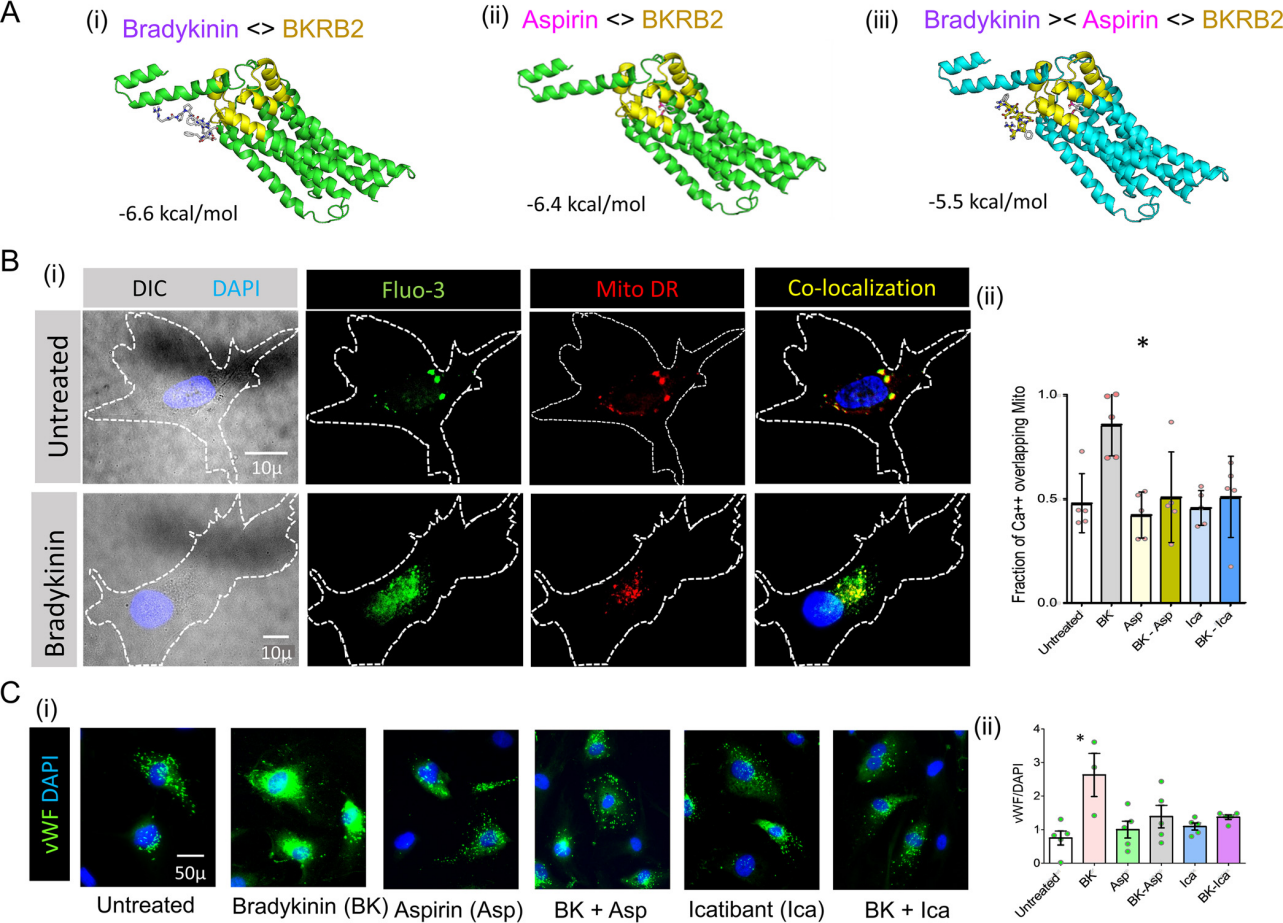
Effects of aspirin (Asp) on the binding affinity of BK to BKRB2, and on BK induced calcium signaling in HAoEC. (A i-ii), Ribbon view and binding affinity (ΔG in kcal/mol) of ED3 and ED4 domain of human BKRB2 with aspirin and with BK in the presence of aspirin respectively as computed using AutoDock Vina molecular docking software. (B-i), High resolution DIC, Fluo-3 (green), Mito Deep Red (MitoDR, red) and co-localization images of HAoEC without or with BK exposure for 15 s. (B i-ii)-Fraction of cytoplasmic Fluo-3 (Ca^++^) overlapping MitoDR fluorescence in HAoEC following BK, Asp-BK, Ica, Ica-BK treatment, or respective control conditions (data from 3 independent experiments, *, *P* < 0.05). (C i-ii) Representative images of HAoEC (left) and MFI values of cytoplasmic vWF in HAoEC and following indicated treatments. (*n* > 300 cells, *, *P* < 0.05).

To better understand BK/BKRB2 binding and the potential to interfere with this interaction, we used open source software ‘Auto Dock Vina’ to estimate a ligand binding affinity score of −6.6 kcal/mol for BK to BKRB2 (Fig. 5A-i) (23). The binding score for Aspirin to BKRB2 was −6.4 kcal/mol (Fig. 5A-ii). We also performed reassessing of this docked structure using alternative software like MOLDOCK and PRODIGY (not shown) (24). Our results, as shown in Table 1, identified aspirin and colchicine as suitable candidate molecules that may disrupt BK/BKRB interactions. Our computational BKBR2 ligand engagement data suggests that aspirin can bind to BKBR2 and decreases BK binding affinity to −5.5 kcal/mol (Fig. 5A-i–iii).

**TABLE 1:** Derived binding energy of aspirin and other molecules to BKRB2 and projected effects on BK binding to BKRB2

Drug	Binding energy to BkRB2 (kcal/mol)	Binding energy of BK to BKRB2 in presence of drug (kcal/mol)
		−6.6
Aspirin	−6.4	−5.5
Naloxone	−8.7	−5.9
Alogliptin	−8.6	−6.3
Colchicine	−7.8	−5.5
Arthemether	−8.2	−6.0
Galantamine	−7.9	−6.0
Apomorphine	−9.0	−5.7
Demeclocycline	−9.4	−6.3
Aztreonam	−7.4	−5.8
Podophyllin	−8.5	−5.8
Morphine	−8.4	−6.3

To assess the effects of BK on endothelial cells and a role for aspirin in disrupting these effects, we stimulated HAoECs with BK in the presence or absence of Aspirin and assessed calcium flux measured by the relative cytoplasmic Ca^++^ localization around mitochondria clusters, labeled respectively by Fluo-3^AM^ and Mito-DR (25). We modified the previously reported dynamic intracellular Ca^2+^ mobilization study protocol to a single time point of 15 s of BK exposure and compared the relative fluorescence intensities expressed as fraction of Ca^++^ fluorescence overlapping the localized areas of mitochondrial fluorescence. Representative images show Fluo-3, Mito-DR, and co-localization in untreated (upper row) and BK exposed (15 s) human aortic endothelial cells. Analyzed data (*n* = 5) are presented in Fig. 5B-i–ii, indicating a significant (*P* = 0.0317) mitochondrial association of Ca^++^ following BK exposure. In these studies, the presence of aspirin or the BKRB2 antagonist icatibant could completely block this BK induced mitochondrial relocation of Ca^++^. The role of aspirin in differential Ca^++^ signaling pattern between the cytosol and the mitochondria suggests that the intracellular Ca^++^ concentrations in these two compartments are modified during BK-induced endothelial cell activation possibly by de-regulating Ca^++^ dependent signaling events which could be protected by icatibant or aspirin.

Next, to elucidate the functional impact of BK exposure on the endothelium and to assess the potential beneficial role of aspirin, we exposed endothelial cells to BK and assessed the surface expression of vWF by immunofluorescence microscopy. In these studies BK induced higher expression of vWF and aspirin or the BKRB2 inhibitor, icatibant (Ica) were able to counteract it (Fig. 5C i–ii). Overall, these findings are indicative of a potential beneficial impact of aspirin in lowering the binding affinity of BK to BKRB2, modifying endothelial cell Ca^++^ mobilization and protecting cells from BK-induced endothelial dysfunction.

## DISCUSSION

In the current study, we investigated mechanistic consequences of the engagement of Wuhan strain SARS-CoV-2 virus spike protein with ACE2+ primary airway epithelial cells and primary aortic endothelial cells. We hypothesize that this modality of virus entry in the broncho-alveolar tree can lead to the onset of ARDS, characterized by pulmonary microvascular thrombosis and capillary leak, in addition to immune cell infiltration and cytokine release (26). In a number of recent publications it have been indicated that SARS-CoV-2 could preferentially enters into vascular ECs directly inducing structural modification prior to migration to different organs and tissues inducing dysfunctional changes and apoptosis (27, 28). However we kept our study limited to using SARS-CoV-2 spike protein only and observed comparable effects.

Our results are consistent with a model, whereby SARS-CoV-2 triggers relative loss of ACE2 cell surface expression and that spike protein alone, may be sufficient to cause this effect. The loss of ACE2 is likely to lead to elevated, local concentrations of BK, which, according to our observations, could induce endothelial cell activation and dysfunction; however, we did not measure BK in COVID-19 in this study. Surprisingly, exposure of HAoECs to spike protein alone induced dysfunction and ultimately led to apoptosis, raising the possibility that spike protein/endothelial cell interactions could directly contribute to vascular pathology in COVID-19.

Several recent studies and data sets both on whole tissue transcriptomics and scRNA-seq have examined ACE2 expression patterns on the mRNA level (6). In a recent study, Hikmet et al. presented an updated systematic evaluation of both ACE2 mRNA and protein expression in a large range of tissues, confirming the expression of ACE2 in several epithelial barrier tissues, although ACE2 expression in the respiratory tract was limited compared to expression in other barrier tissues (3). Our observations on normal lung samples indicate substantial surface expression of ACE2 in the respiratory tract, pointing the high susceptibility of human airway epithelia to SARS-CoV-2 infection. We compared the heterogeneity of ACE2 expression in the alveolus, and extensive expression of BKRB2 in lung sections. The proximity and occasional overlap of ACE2+ and BKRB2+ cells in the respiratory tract highlights the potential consequences of reduced ACE2 expression that could lead to localized elevation in BK expression, and activation of BKRB2+ cells in the airway.

We observed that there was an overall significant reduction in ACE2 levels measured along the lining of alveoli in tissues from COVID-19 patients in sharp contrast to that of normal lung. Importantly, due to limited tissue availability, we were unable to obtain well-matched controls (age and sex) for these analyses. Nonetheless, this observation is consistent with a model whereby direct infection by SARS-CoV-2 in the lower respiratory tract could lead to diminished ACE2. In previous reports, SARS-CoV-2 virus reduced cell surface expression of ACE2 and worsened experimentally induced lung inflammation (29, 30). Our *in vitro* studies here suggest that engagement of ACE2 with spike protein alone, without the cellular damage caused by viral replication, is sufficient to reduce cell surface ACE2 expression. Although we did not define the mechanism of ACE2 downregulation, binding of SARS-CoV-2 spike protein to ACE2, is proposed to induce ACE2 shedding from the cell surface, and evidence has been presented that this process is required for cellular uptake of the virus (14).

In addition to the implications of ACE2 downregulation, our observations also point to a novel effect of spike protein on endothelial cell function and stability. We documented that the engagement of spike protein reduces surface ACE2 and intracellular KLF2 expression, while increasing cell surface expression of vWF in primary human arterial endothelial cells. The changes in KLF2 and vWF expression are indicative of endothelial dysfunction and may perpetuate vascular inflammation and coagulation (31, 32).

We also found that exposure to SARS-CoV-2 spike protein induced caspase activation and apoptosis in endothelial cells in a dose and time dependent manner. The molecular mechanism accounting for this effect and the extent to which this might occur *in vivo* are unknown. Nonetheless, the potential loss of endothelial cell integrity and the subsequent activation of the contact activation system by a negatively charged bare sub-endothelial surface could also lead to activation of the coagulation cascade as well as BK generation (33, 34). Endothelial activation, detachment, and apoptosis could be possible triggering mechanisms for coagulopathies and the “cytokine storm” linked to COVID-19 pathogenesis (35).

There is an increasing number of published reports concerning the clinical course of COVID-19 which are startlingly different form previous medical knowledge. Spanning the last 2 decades, research on the pathogenesis of SARS corona virus extensively focused on the RAS and it was later speculated that SARS-CoV-2 infection potentially provoked a similar pathogenesis involving RAS (36). The other major role of ACE2, however, centers on regulation of the kinin-kallikrein systems (37, 38). As part of its enzymatic function, ACE2 is one of the potential inactivating factors responsible for neutralizing about 85% of the circulating BK in the pulmonary vascular bed, contributing to one of the most important nonrespiratory functions of the lungs (8, 39). Moreover, recent human data indicates that ACE2 protects against pulmonary capillary fluid leaks, otherwise induced by BK (40). BK that has a half-life of about 17 s in the blood is an acute phase mediator of inflammation, pain, and is a highly vasoactive agent owing to the presence of BKRB2 in the endothelial cell surface (Fig. S3A in the supplemental material) (8). BK otherwise facilitates capillary dilation and leaking of exudative fluid from blood by decreasing the expression of tight-junction forming E-cadherins in endothelial cells (41, 42). In inflammatory settings, BK can further facilitate neutrophil and possibly other immune cells recruitment into the pulmonary interstitial tissue, and promote leakage of protein rich fluid into the peri-alveolar space (42). Thus, a model for the triggering mechanism of ARDS and associated cardio-respiratory instability in the hyper acute cases of COVID-19 would be direct involvement of the virus causing decreased ACE2 availability and subsequent reduction on BK degradation via spike protein-dependent interactions, leading to increased downstream BK signaling in the endothelial cells via BKRB2, alveolar capillary leak, and infiltration of activated immune cells (43). In addition, downregulation of ACE2 can occur in the presence of other co-morbid pathogen-derived ligands, since in mice, infection of lungs with bacteria or exposure of lungs to endotoxin, results in ACE2 downmodulation and neutrophil lung infiltration (29). In animal models, SARS-CoV infection activated the complement pathway, suggesting that complement signaling contributes to disease pathogenesis in the lung as well as systemic inflammation (44). Complement inhibitors have recently shown some promise in a recent clinical trial with a small number of critically ill COVID-19 patients (45). Moreover, the intravascular innate immune system, that includes the contact, complement, coagulation, and fibrinolysis systems, is also deeply associated with the pathogenesis of ARDS in SARS-CoV-2 infection (46). Furthermore, other viral infections such as those caused by Maripa virus (Hanatvirus), have been described with presenting symptoms similar to those of SARS-CoV-2 infection, implicating pulmonary capillary leak and rapidly progressing ARDS (47). Distinguishing the relative importance of Spike/ACE2 interactions from other mechanisms that likely contribute to ARDS, is an important consideration for future pathogenesis studies.

**FIG 6:**
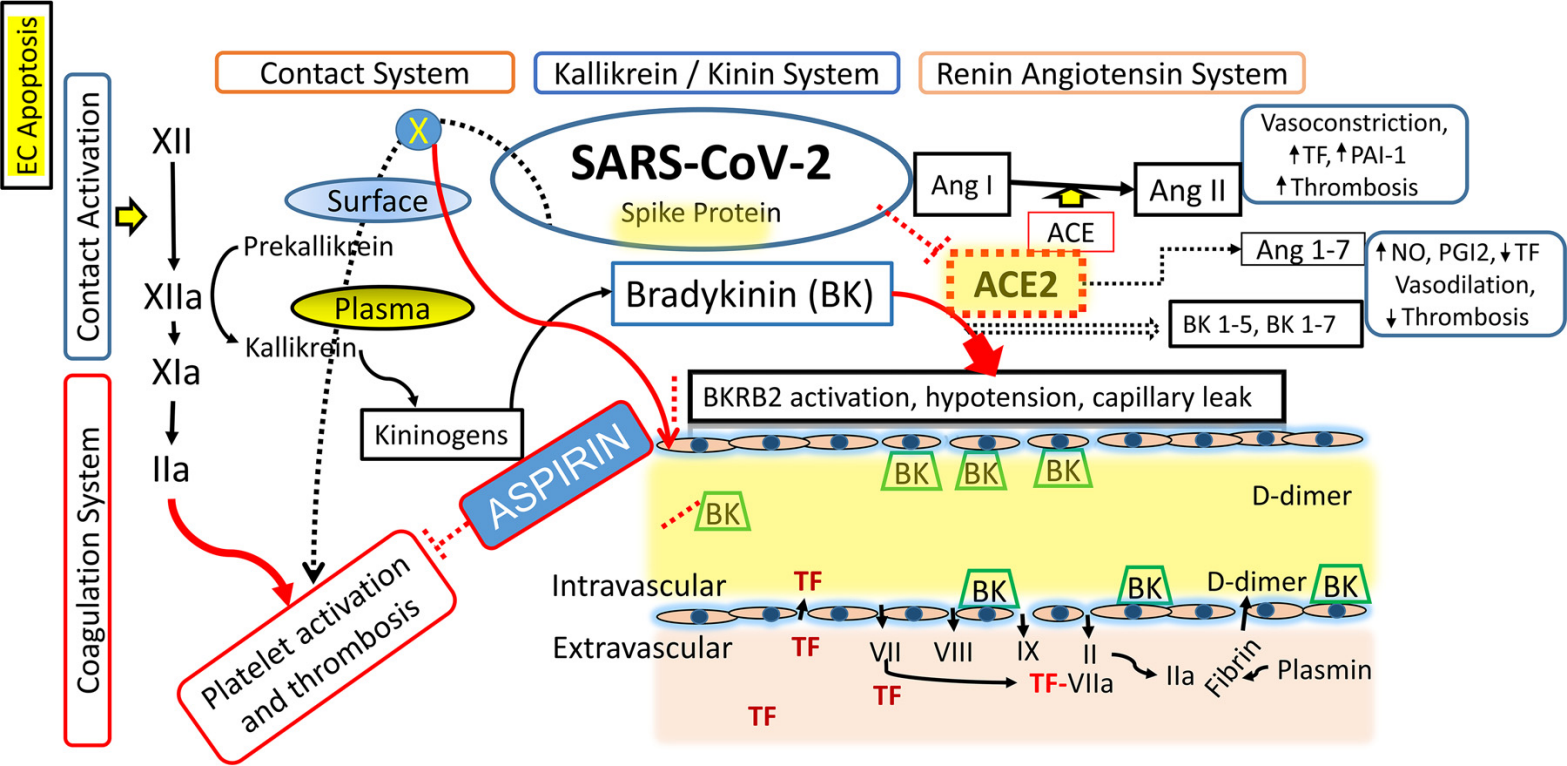
Schematic diagram showing triggering of contact activation system by a negatively charged surface leads to activation of the coagulation cascade and bradykinin (BK) generation. BK binds to the BKRB2 on endothelial cells, increases plasma coagulation parameters secondary to increased alveolar capillary permeability in and extravascular coagulation.

The serine protease tissue kallikrein-1 generates kallidin (Lys-BK) mainly from circulating low-molecular-weight kininogen, whereas plasma kallikrein, a component of the contact activation system, generates BK from high-molecular-weight-kininogen (HK) (48) (Fig. 6). Both peptides (BK and Lys-BK) are equipotent agonists of the preformed endothelial B2 receptor for BK (BKRB2) and these interactions can influence endothelial barrier function, leading to changes in fluid and cellular permeability at the level of cardiac and pulmonary microvasculature (49). While ACE is the major kinin-destroying peptidase in the extracellular fluid (50) ACE inhibitor-induced angioedema is reportedly reversed by BKRB2 antagonist icatibant (51–53). The conventional nonsteroidal anti-inflammatory agent aspirin has been recognized as an allosteric inhibitor of BKRB2. According to previous reports, the BKRB2, a member of the G protein-coupled receptor superfamily, which is involved in a variety of physiological functions, including vasodilation, electrolyte transfer in epithelium, mediation of pain, and inflammation, could be competitively blocked by aspirin both *ex-vivo* and in the clinics (17, 18). In these experiments, aspirin could lower the B2 receptor ligand binding affinity and accelerate the dissociation rate of bradykinin–receptor complexes (17). Consistent with these findings and other reports showing a link between BKRB2 receptor activation and calcium mobilization, we found that aspirin reduces BK-induced mitochondrial mobilization in primary endothelial cells and reduces BK-mediated endothelial cell dysfunction *in vitro* at therapeutic concentrations. Our observations provide rationale for considering inhibition of BK activity as an early intervention strategy in COVID-19.

## MATERIALS AND METHODS

### Antibodies and reagents.

The following antibodies were used for all of the fluorescent histology and immunofluorescence studies: anti-human mouse primary monoclonal antibodies toward ACE2 (R&D Systems; MAB933, clone 171606), BKRB2 (R&D Systems; MAB47251, clone 471916), affinity purified rabbit polyclonal cleaved PARP-1 (Asp214) antibody (Cell Signaling Technology, Inc.; #9541), rabbit polyclonal anti-eNOS (AbCam: phospho-S1177, ab75639), rabbit polyclonal anti-KLF2 (LifeSpan BioSciences; LS-B4570), and antibody raised against peptide spanning amino acids 216–265 of human KLF2 (Q9Y5W3; National Center for Biotechnology Information reference sequence NP_057354.1). Rabbit polyclonal pro-surfactant Protein C Antibody (Novus Biologicals, NBP1-60117). We obtained purified recombinant COVID-19 (isolate Wuhan-Hu-1) protein (YP_009724390.1) (Arg319-Phe541) from Creative Biomart NY, which was fused to His-tagged and expressed in HEK293 cells (Creative Biomart NY; nCoVS-125V COVID-19S). In addition, we used recombinant SARS-CoV-2 Spike protein (active trimer) from R&Dsyetems (#10549-CV-100) in some experiments. We purchased following reagents for various experiments as indicated in the later sections: Aspirin (Sigma-Aldrich #A2093), BK acetate salt (Sigma-Aldrich #90834), Icatibant acetate (Cayman Chemical Company #24083); Fluo-3 AM (Cayman Chemical Company #14960), MitoTracker Deep RedFM (Molecular Probes: M22426), and SR-FLICA Poly Caspase assay kit (ImmunoChemistry Technologies LLC., MN, USA).

### Pulmonary tissues.

Healthy pulmonary tissue from autopsy samples were purchased from commercial sources as formaldehyde fixed paraffin embedded tissue micro-array slides (US Biomax, Inc., MD, USA). In addition, postmortem lung tissue FFPE slides were obtained from four patients with COVID-19, who were seen at University Hospitals of Cleveland. De-identified lung tissue samples were obtained using protocols and procedures that had been approved by University Hospitals of Cleveland Institutional Review Board.

### Cells and *in vitro* experiments.

Primary human endothelial cells, lung fibroblasts, and HAEpCs, and the human intestinal cell line CACO-2 were used for this study. The HAoECs were purchased from PromoCell, propagated, and cultured in glass slide chambers (Millicell EZ; Millipore) with EGM-MV (PromoCell), according to the manufacturer's instructions. All experiments were performed using HAoECs between the seventh and tenth passages. At the end of treatment, HAoECs were processed for immunofluorescence microscopy as indicated and examined. The human lung fibroblast cell line MRC-5 (ATCC CCL-171) was purchased from ATCC, propagated, and handled according to ATCC specified protocol and medium. Primary human alveolar epithelial cells (ABC-TC3770) were purchased from Accegen Biotechnology and propagated/used for specific experiments as per specified protocol and supplied epithelial cell growth medium (ABM-TM3770). The human colon adenocarcinoma derived intestinal cell line CACO-2 (86010202) was purchased from Milipore-Sigma and was used as specified.

### Immuno-fluorescence microscopy and image analysis.

Tissue micro array slides (RS321, LCN24, and LC561; US Biomax, Inc., Derwood, MD) that included nonpathological pulmonary tissue samples from multiple donors was used for tandem immunofluorescence staining with three primary antibodies and sequential application of tyramide tagged fluorophores for epi-fluorescence microscopy. Briefly, the deparaffinized sections were re-hydrated, treated with 3% hydrogen peroxide solution to inhibit endogenous peroxidase, and processed at 95°C for 20 min in 10m sodium citrate, 0.05% Tween 20, pH 6.0 for epitope retrieval, followed by blocking with 2% BSA in TBST (TBS + Triton X-100 0.025%) for 1 h at room temperature. Indicated primary antibodies were diluted (1:50) with 2% BSA in TBST. Sections were subjected to 2 tandem rounds of staining with each primary antibody followed by a secondary HRP-conjugated polymer. Tyramide signal amplification technology was applied for sequential labeling of ACE2, and BKRB2 identified by - Cy3 (red)/CF®488A (green) –tyramide (Biotium, Inc., Fremont, CA) fluorochrome following stringent protocol as specified elsewhere (54). The slide was mounted in Vectashield with DAPI (Vector laboratories, Burlingame, CA) for epi-fluorescence microscopy using 20X, and 100X oil immersion objectives. Iterative deconvolution module (up to 10 iterations) of the ImageJ software (NIH) was used to optimize some of the acquired digital images for presentation. A second slide was stained in the same manner as above except that isotype controls were substituted for anti-ACE2 and anti-BKRB2 primary antibodies.

The stained tissue array slides RS321, and LCN24, were imaged by epi-fluorescence microscopy using 20X, and 100X oil immersion objectives. ImageJ software (http://imagej.nih.gov/ij/) was used to analyze the acquired digital images of respective control and COVID-19 lung tissues. Briefly, images of each fluorescence channel were converted to 8-bit monochrome images followed by background correction. The representative numerical fluorescence intensity values were measured for ACE2, and BKRB2. We analyzed and presented the normalized MFI values of the indicated targets using the software GraphPad Prism version 6.0 as a measurement of their relative expression (55). We also applied iterative deconvolution methods (up to 10 iterations) to enhance and study high-resolution images.

### Detection of *in situ* caspase activation in endothelial cells by fluorochrome-labeled caspase inhibitor (SR-FLICA caspase assays).

Pan-caspase activation in human aortic endothelial cells following indicated treatments were measure by fluorochrome-labeled caspase inhibitor SR-FLICA according to protocol specified by the manufacturer (https://immunochemistry.com/product/sr-flica-poly-caspase-assay-kit/). Primary human aortic endothelial cells grown on sterile 8 chamber slide, were first incubated with or without BK, staurosporine (positive control), SARS-CoV-2 spike protein (SpkPr), and SR-FLICA reagent as indicated. Apoptotic endothelial cells were stained with SR-FLICA on living cells, which require periodic maintenance and cultivation several days in advance. After an incubation of 15 min at room temperature in the dark, the microscopy slides were briefly rinsed with PBS and immunostained with mouse anti-actin primary and FITC conjugated anti-mouse secondary antibodies in sequence after blocking for nonspecific antibody binding. Next, the chamber slide were air dried and mounted in Vectashield with DAPI.

### Detection of Cleaved PARP-1 by Western blotting.

Human aortic endothelial cells were stimulated with indicated agonists for 72 h, lysed at 4°C in a lysis buffer containing protease and phosphatase inhibitors, briefly centrifuged, and supernatants were mixed with equal volume of a SDS-PAGE sample buffer containing DTT. Equal amounts of protein (∼50 μg) were separated on a 10% Tris-HCl gradient gel and transferred to PVDF membranes for immunodetection of cleaved PARP-1 by a LI-COR system.

### Evaluation of calcium signaling in primary human endothelial cells.

HAoECs grown on sterile 8 chamber slides, were first incubated with or without aspirin, FCCP (data not shown) and (i) Fluo-3 AM (Cayman Chemical, MI, USA) 5 μM and (ii) Mito-Deep-Red 500 nM for 15 min in a buffer containing 140 mM NaCI, 5 mM KCI, 1 mM MgCl_2_, 1 mM CaCl_2_, 10 mM glucose, and 10 mM Na HEPES. Following a subsequent 15 s exposure to BK (10 μM) to the indicated groups, cells were then washed 3 times with the same HEPES buffer (25). The cells were subsequently fixed in 100% ice-cold methanol for 15 min at −20°C and rinsed 3 times with PBS for 5 min. Next, the chamber slide were air dried and mounted in Vectashield with DAPI. The quantitative measurements of cytoplasmic Ca^++^ mobilization and mitochondrial localization in individual endothelial cells was documented using a table-top epi-fluorescence microscope (EVOS FL Auto Imaging System, Life Technologies, USA) equipped with an LED light cubes. Cells were viewed with a 100x oil immersion objective. The optimized application of image thresholding module in ImageJ software was applied to specify region of interest (RIO) defining Ca^++^ enriched zonesandmitochondria.

### Computational evaluation of ACE2 and BKRB2 ligand binding.

Interactions of the spike glycoprotein of SARS-CoV-2 with the human ACE2 receptor were studied using virtual molecular docking. The virtual docking studies were initially performed using ZDOCK software (56). Subsequently, these the molecular docking calculations were verified using AutoDock Vina software suit (23). AutoDock Vina designed to accept coordinate files for receptor and ligand and predict optimal docked conformations via stochastic search methods. Because of the unavailability of the crystallographic structure of BKRB2, the 3D atomistic model of BKRB2 was built by homology modeling SEAGrid (http://www.seagrid.org), which is acknowledged for computational resources and services for the selected results used in this publication (45, 57, 58).

### Statistics.

Values are expressed as means ± standard errors of mean. Comparisons between unrelated groups used nonparametric two-tailed Mann-Whitney U tests. Paired group analyses used Wilcoxon matched pairs signed rank test. All statistics were performed using Prism 6 software (GraphPad). Differences were considered statistically significant if the *P value* was less than 0.05.

## Supplementary Material

Reviewer comments
