# Supplementary material for: SARS-CoV-2 Spike Protein Destabilizes Microvascular Homeostasis
Source: Microbiol Spectr. 2021 Dec 22;9(3):e00735-21. doi: 10.1128/Spectrum.00735-21 (PMC8693925; doi:10.1128/Spectrum.00735-21)
Supplement: SUPPLEMENTAL FILE 1 — Supplemental material. Download SPECTRUM00735-21_Supp_1_seq11.pdf, PDF file, 0.3 MB [file spectrum00735-21_supp_1_seq11.pdf]

SUPPLEMENTARY FIGURES

FIGURE S1

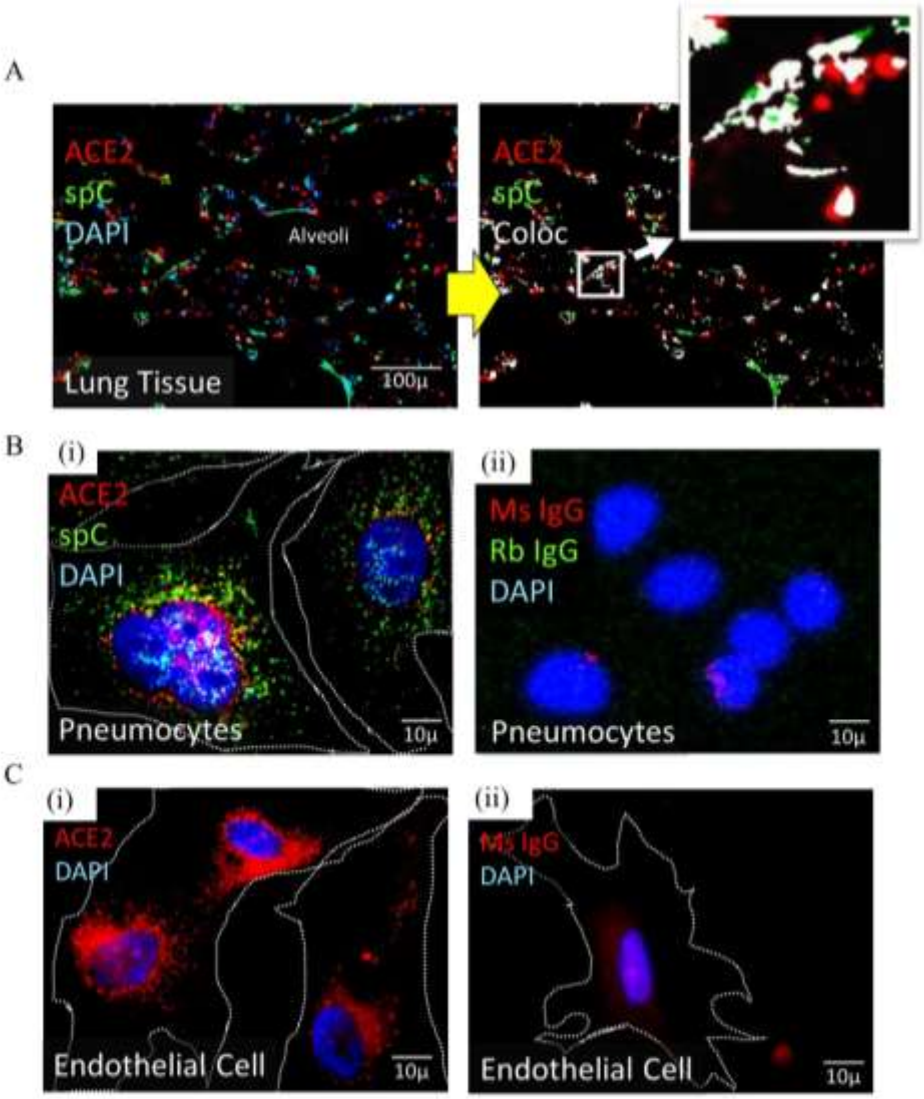

Figure S1 Interaction of SARS-CoV-2 spike protein with ACE2, in human pulmonary alveolar epithelial cells. A: low resolution epi-fluorescent microscopy image of normal lung tissue showing ACE2 (red), surfactant protein C (spC, green) and DAPI (left panel) and colocalized points (coloc:white) in the right panel. B-i, high resolution immuno-fluorescence images of ACE2, spC and DAPI in cultured primary alveolar epithelial cells (middle), and B-ii, respective control images using antibody isotypes in the same experiment. C, high resolution image of 3 primary human aortic endothelial cells showing representative DIC (BW: C-i), ACE2/DAPI (red/blue: C-ii), and respective control image using ACE2 mouse IgG antibody isotypes in the same experiment (red/blue: C-iii).

FIGURE S2

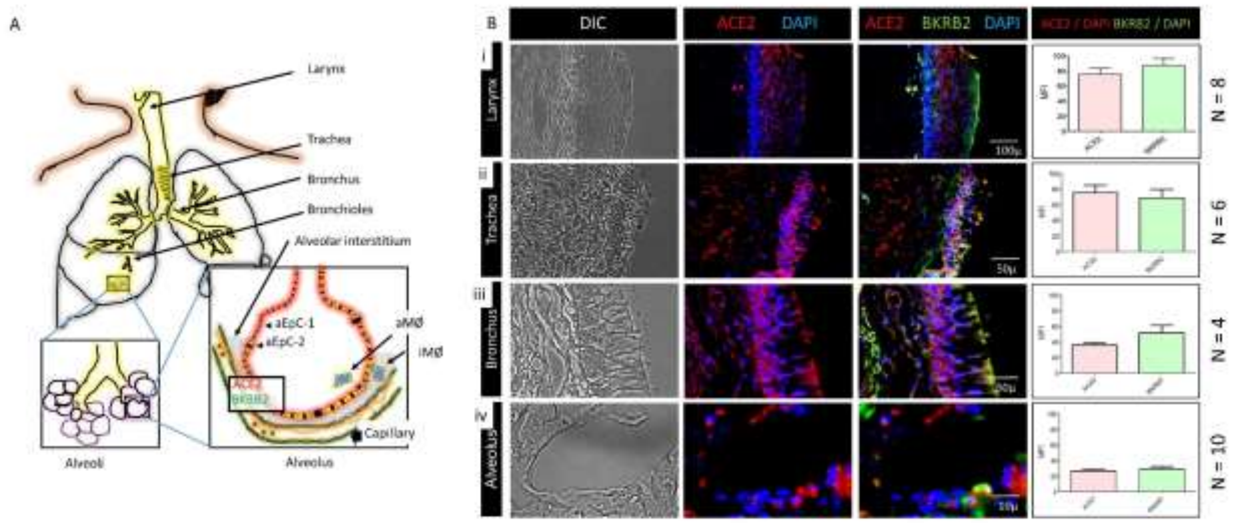

Figure S2 Differential expression of ACE2 on the pulmonary bronchial tree and alveolar epithelium. A. Schematic diagram showing pulmonary airway and patterns indicating the distribution of alveolar type-1 (aEpC-1) and type-2 (aEpC-2) epithelial cells surface expressions of ACE3 and BKRB2. B. Representative DIC (BW), ACE2/DAPI (red/blue), ACE2/BKR2/DAPI (red/green/blue) epi-fluorescent microscopy images and mean fluorescence intensity (MFI) of ACE2 and BKRB2 from indicated numbers of healthy pulmonary tissue samples shown in left to right columns. Images and MFI values from larynx (B-I, n = 8)), trachea (B-ii, n = 6), bronchus (B-iii, n = 4), and alveolus (b-iv, n = 10) are shown in respective rows in descending order.

FIGURE S3

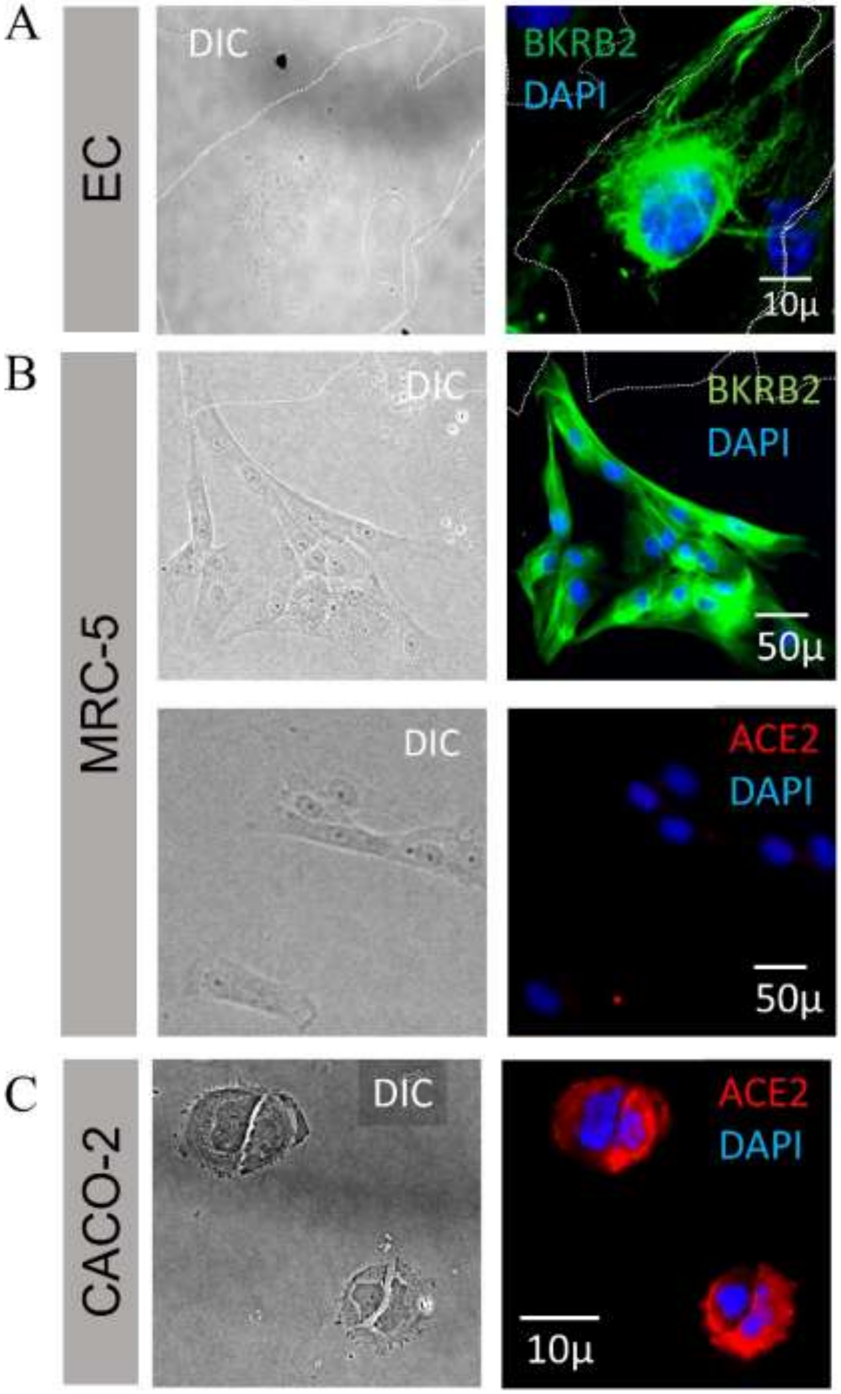

Figure S3 Protein expressions of ACE2 and Bradykinin receptor B2 (BKRB2). High resolution immunofluorescence images of (A.) human endothelial cells (EC): left panel – DIC image and right BKRB2 (green), (B.) intestinal epithelial cells (CACO-2): left panel – DIC and right ACE2 (red), and (C.) pulmonary alveolar fibroblast (MRC-5): left panel – DIC image and right RKRB2 (green-upper row, and right ACE2 (red-lower row).

Table S1

Subject	Age (Years)	Gender	Tissue (Autopsy)
Cont 1	26	M	lung
Cont 2	50	M	lung
Cont 3	49	M	lung
Cont 4	55	M	lung
Cont 5	35	M	lung
Cont 6	48	M	lung
Cont 7	38	F	lung
Cont 8	25	M	lung
Cont 9	22	M	lung
Cont 10	35	M	lung
#1 COVID-19	81	M	lung
#2 COVID-19	54	M	lung
#3 COVID-19	64	F	lung
#4 COVID-19	49	F	lung

Age, and gender distribution of control and COVID-19 positive human pulmonary tissues used for the study.
