# Supplementary material for: SARS-CoV-2 Spike Protein Destabilizes Microvascular Homeostasis
Source: Microbiol Spectr. 2021 Dec 22;9(3):e00735-21. doi: 10.1128/Spectrum.00735-21 (PMC8693925; doi:10.1128/Spectrum.00735-21)
Supplement: Reviewer comments [file reviewer-comments.pdf]

AMERICAN SOCIETY FOR MICROBIOLOGY | Microbiology Spectrum

SARS-CoV-2 Spike Protein Destabilizes Microvascular Homeostasis

Soumya Panigrahi, Tamal Goswami, Brian Ferrari, Christopher Antonelli, Douglas Bazdar, Hannah Gilmore, Michael Freeman, Michael Lederman, and Scott Sieg

Corresponding Author(s): Scott Sieg, Case Western Reserve University

Review Timeline:

Submission Date:	August 25, 2021
Accepted:	November 6, 2021

Editor: Takamasa Ueno

Reviewer(s): The reviewers have opted to remain anonymous.

Transaction Report:

(Note: With the exception of the correction of typographical or spelling errors that could be a source of ambiguity, letters and reports are not edited. The original formatting of letters and referee reports may not be reflected in this compilation.)

DOI: https://doi.org/10.1128/Spectrum.00735-21

November 6, 2021

Dr. Scott F. Sieg
Case Western Reserve University
Medicine/Division of Infectious Diseases
Wood Blg., Rm. 119
2109 Adelbert Road
Cleveland, Ohio 44106-4984

Re: Spectrum00735-21 (SARS-CoV-2 Spike Protein Destabilizes Microvascular Homeostasis)

Dear Dr. Scott F. Sieg:

Your manuscript has been accepted, and I am forwarding it to the ASM Journals Department for publication. You will be notified when your proofs are ready to be viewed.

The ASM Journals program strives for constant improvement in our submission and publication process. Please tell us how we can improve your experience by taking this quick Author Survey.

As an open-access publication, Spectrum receives no financial support from paid subscriptions and depends on authors' prompt payment of publication fees as soon as their articles are accepted. You will be contacted separately about payment when the proofs are issued; please follow the instructions in that e-mail. Arrangements for payment must be made before your article is published. For a complete list of **Publication Fees**, including supplemental material costs, please visit ourwebsite.

Corresponding authors may join or renew ASM membership to obtain discounts on publication fees. Need to upgrade your membership level? Please contact Customer Service at Service@asmusa.org.

Thank you for submitting your paper to Spectrum.

Sincerely,

Takamasa Ueno
Editor, Microbiology Spectrum

Journals Department
American Society for Microbiology
1752 N St., NW
Washington, DC 20036
E-mail: spectrum@asmusa.org

Supplemental Material: Accept
